# Current State and Future Prospects of EEG and fNIRS in Robot-Assisted Gait Rehabilitation: A Brief Review

**DOI:** 10.3389/fnhum.2019.00172

**Published:** 2019-06-05

**Authors:** Alisa Berger, Fabian Horst, Sophia Müller, Fabian Steinberg, Michael Doppelmayr

**Affiliations:** ^1^Department of Sport Psychology, Institute of Sport Science, Johannes Gutenberg-University, Mainz, Germany; ^2^Department of Training and Movement Science, Institute of Sport Science, Johannes Gutenberg-University, Mainz, Germany; ^3^Centre for Cognitive Neuroscience, University of Salzburg, Salzburg, Austria

**Keywords:** multi-modal approach, electroencephalography, functional near-infrared spectroscopy, robot-assisted gait training, motor recovery, neurorehabilitation, brain-machine interface, brain stimulation

## Abstract

Gait and balance impairments are frequently considered as the most significant concerns among individuals suffering from neurological diseases. Robot-assisted gait training (RAGT) has shown to be a promising neurorehabilitation intervention to improve gait recovery in patients following stroke or brain injury by potentially initiating neuroplastic changes. However, the neurophysiological processes underlying gait recovery through RAGT remain poorly understood. As non-invasive, portable neuroimaging techniques, electroencephalography (EEG) and functional near-infrared spectroscopy (fNIRS) provide new insights regarding the neurophysiological processes occurring during RAGT by measuring different perspectives of brain activity. Due to spatial information about changes in cortical activation patterns and the rapid temporal resolution of bioelectrical changes, more features correlated with brain activation and connectivity can be identified when using fused EEG-fNIRS, thus leading to a detailed understanding of neurophysiological mechanisms underlying motor behavior and impairments due to neurological diseases. Therefore, multi-modal integrations of EEG-fNIRS appear promising for the characterization of neurovascular coupling in brain network dynamics induced by RAGT. In this brief review, we surveyed neuroimaging studies focusing specifically on robotic gait rehabilitation. While previous studies have examined either EEG or fNIRS with respect to RAGT, a multi-modal integration of both approaches is lacking. Based on comparable studies using fused EEG-fNIRS integrations either for guiding non-invasive brain stimulation or as part of brain-machine interface paradigms, the potential of this methodologically combined approach in RAGT is discussed. Future research directions and perspectives for targeted, individualized gait recovery that optimize the outcome and efficiency of RAGT in neurorehabilitation were further derived.

## Introduction

According to the World Health Organization, over one billion people are affected by gait and balance impairments due to neurological diseases that impact independent living and quality of life (Stump, 2007; Turner et al., 2013; Calabrò et al., 2016). With respect to maladaptive brain functions in the motor domain (e.g., following stroke), the brain has the remarkable capacity to repair itself to a certain extent. Such processes occur through functional and structural changes during motor (re-)learning, which are collectively termed neuroplasticity (Pascual-Leone et al., 2005; Pascual-Leone et al., 2011; Kays et al., 2012; Chen et al., 2015). In recent years, robot-assisted neurorehabilitation has been applied in addition to manual-assisted therapy due to its provision of early, intensive, task-specific, and multi-sensory training, which is thought to be most effective for motor recovery by favoring neuroplastic changes (Turner et al., 2013; Calabrò et al., 2016). Indeed, current literature suggests that patients with neurological diseases improved their walking ability (Calabrò et al., 2016), walking speed (Benito-Penalva et al., 2012; Uçar et al., 2014), leg muscle force (Beer et al., 2008), step length, and gait symmetry (Husemann et al., 2007; Esquenazi et al., 2013) due to robotic rehabilitation. Nevertheless, the science-based evidence for improved gait recovery is mixed due to heterogeneous study designs and methods, coupled with a lack of knowledge regarding the underlying neurophysiological mechanisms of robot-assisted gait training (RAGT) (Swinnen et al., 2010, 2015; Knaepen et al., 2015).

New insights into brain activity during RAGT would allow the improvement of robotic rehabilitation, however, it is a challenge because of movement-related signal artifacts arising during the interaction of robot systems with their users. In contrast to stationary neuroimaging methods such as functional magnet resonance imaging (fMRI) which do not allow the analysis during movements (Gramann et al., 2011), wearable mobile brain imaging (MoBI) systems provide an approach to fully inv estigate how natural, varying behavior is guided by complex neural dynamics (Makeig et al., 2009). EEG and fNIRS are non-invasive, portable, and cost-effective methods representing the MoBI approach by enabling the measurement of brain activity in natural environments.

Electroencephalography records the integrated and synchronized activity of pyramidal neurons in the cerebral cortex, either postsynaptic potentials associated with neural activation [e.g., event-related potentials (ERPs)] or changes and strengths of various oscillation frequencies (i.e., delta, theta, alpha, mu, beta, and gamma) which are expressed as power spectral density or coherence (Knaepen et al., 2015). In addition to an excellent temporal resolution, reasonable spatial accuracy can be ensured by new high-density systems (Robinson et al., 2017) or statistical methods such as independent component analysis (ICA) decompositions by reconstructing the origin of EEG activity (Makeig et al., 2004; Onton et al., 2006; Gramann et al., 2010). Furthermore, artifacts during walking due to head movements, eye movements or neck muscle activity can be detected with ICA (Gwin et al., 2010, 2011; Wagner et al., 2012; Snyder et al., 2015) or artifact removal methods including moving average and wavelet-based techniques (Kline et al., 2015), thus, resulting in improved signal-to-noise ratio. Studies investigating brain oscillations during walking (Petersen et al., 2012; Severens et al., 2012; Solis Escalante et al., 2012; Wagner et al., 2012, 2016; Sanctis et al., 2014; Seeber et al., 2014; Bulea et al., 2015) showed sustained mu and beta desynchronization within sensorimotor cortex (SMC; Severens et al., 2012; Wagner et al., 2012, 2014; Seeber et al., 2014; Bulea et al., 2015) demonstrating that motor cortex and corticospinal tract contribute directly to the muscle activity in locomotion (Petersen et al., 2012).

In contrast to EEG, fNIRS relies on the principle of neurovascular coupling measuring changes in regional cerebral blood flow, oxygenated hemoglobin (Hboxy), and deoxygenated hemoglobin (Hbdeoxy) induced by neuronal activation, which is analogous to the blood-oxygenation-level-dependent responses measured by fMRI (Ferrari et al., 2004). Both provide information regarding the spatial location of the recorded activity, whereas the temporal resolution is limited due to the intrinsically slow processes of hemodynamic changes. Compared to fMRI, fNIRS is – although with lower resolution (Koch et al., 2008) affordable and easily implementable in a portable system, thus facilitating a wider range of applications. Recently, wearable and wireless systems were developed which allow the topographical representation of hemodynamic responses over the cortical surface due to high-density multi-distance channels (Shin et al., 2017). Furthermore, they are lightweight and robust to motion artifacts due to the absence of fiber-optic bundles (Pinti et al., 2018). Despite advantages and developments, the challenge remains to distinguish physiological changes through brain activity from noise and artifacts. Various methods have been proposed recently to correct for motion artifacts, including principle component analysis, spline interpolation, Kalman filtering, wavelet filtering and correlation-based signal improvement, with the result that wavelet filtering seems be the most promising and powerful technique for motion artifact correction in fNIRS data (Brigadoi et al., 2014). Furthermore, new short channels systems enable the detection of scalp-hemodynamics contaminating the fNIRS signal which can be removed from cerebral hemodynamics of long-channels using general linear model to estimate brain activity (Sato et al., 2016). In studies investigating brain activity in terms of Hboxy/Hbdeoxy concentration changes during walking, healthy people had significantly increased brain activity in SMC, premotor cortex (PMC), supplementary motor area (SMA) as well as prefrontal cortex (PFC) (Miyai et al., 2001; Hamacher et al., 2015). In comparison, patients with neurological diseases and elderly people showed increased PFC activity associated with low gait capacity (Harada et al., 2009; Stuart et al., 2018). A standardized method to identify and reduce motion as well as physiological artifacts (e.g., heart rate, mayer waves, respiration) during walking is missing so far (Vitorio et al., 2017).

Based on the independence between electrical and optical measurements as well as the assets and drawbacks of each neuroimaging technique, the multi-modal integration of EEG-fNIRS can partially overcome the limitations encountered by each individual modality (Merzagora et al., 2009; Muthalib et al., 2013), which may help to more accurately characterize the functional activity of neural networks involved in robotic rehabilitation. However, although RAGT is the most frequent robot-assisted intervention for neurological injuries, little is known regarding the neural correlates of gait recovery in RAGT (Kim et al., 2016). In order to determine optimal training parameters for individualized gait therapy protocols, it is essential to understand how robotic devices interact with their users, and thus, how both locomotor control and gait recovery is characterized by brain signals (Solis Escalante et al., 2012; Youssofzadeh et al., 2014, 2016).

Therefore, we (1) surveyed neuroimaging studies focusing on RAGT (summarized in Table 1) with the aim to contribute to the understanding of neurovascular coupling phenomena and (2) inferred the potential of multi-modal integration of EEG-fNIRS in robotic gait rehabilitation (see section Future Prospects for Fused EEG-fNIRS in RAGT).

**TABLE 1:** Overview of studies investigating the neurophysiological mechanisms underlying RAGT using EEG and fNIRS.

**Author(s)**	**Title**	**Robotic system**	**Method**	**EEG/fNIRS device**	**Parameter**	**Sample**	**Outcome**	**Conclusion**
Calabrò et al.,2018	Shaping neuroplasticity by using powered exoskeletons in patients with stroke: a randomized clinical trial	Ekso^TM^	Prospective, pre-post, randomized clinical trial; 1st group: EGT and OGT; 2nd group: OGT; Study design: 45 min per session, five times a week over 8 weeks	EEG Brain Quick SystemPLUS (Micro-med; Mogliano Veneto, Italy); 21 electrodes Sampling rate: 512 Hz; Electrode placement: 10–20 system; Additional: sEMG & TMS	EEG: FPEC EMG: gait performance based on the 10 m walking test, gait cycle, muscle activation muscles; TMS: CSE and SMI from both M1	Stroke patients; Hemiparesis caused by stroke in a chronic phase; Number of participants: 40 patients, 20 each in a group; Age: ≥55 years	FPEC: significant strengthening of EGT group; CSE: significant improvement in EGT group on the affected side; SMI: significant improvement in EGT group on affected side; 10 m walk test: significant improvement in EGT group; General walking quality: significant improvement; Hip and knee muscle activation: significant improvement	EGT seems, in addition to OGT, also promising in gait rehabilitation for patients after a stroke. The study suggests that Ekso could be useful to promote the mobility of people with stroke, thanks to the mechanisms of brain plasticity and remodulation of connectivity that are specifically carried along by the robotic system compared to conventional OGT.
Contreras-Vidal et al.,2018	Neural Decoding of Robot-Assisted Gait during Rehabilitation after Stroke	H2 Exoskelett	Study design: 12 training sessions, 4 weeks Meanwhile, no additional therapy; Walking speed: preset and comfortable for the test persons, could be adjusted while walking	EEG BrainAmpDC, Brain Products, Germany; 64 electrodes Sampling rate: 1000 Hz	Changes in power spectra for the 0.1–3 Hz (delta) band; Additional: walking speed, walking distance	Stroke patients Chronic hemiparesis after a stroke Number of test persons: 5; Exclusion: 1	Suppression of the delta frequency when walking in the occipital scalp area. Increase in delta frequency suppression in the frontal and central scalp regions. Improvement of walking distance and walking speed, which correlated with increased accuracy of offline decoding.	Proof of the feasibility of neuronal decoding of gait kinematics from the EEG during RAGT in chronic stroke patients. Since motor intention recognition from EEG signals is synchronized with motion feedback generated by exoskeleton-assisted movements of the lower extremities, the BMI-H2 system can promote brain reorganization through motor learning, presumably due to activity-dependent brain plasticity. First step in the development of a brain-machine interface to control driven exoskeletons.
Knaepen et al.,2015	Human-Robot Interaction: Does Robotic Guidance Force Affect Gait-Related Brain Dynamics during Robot-Assisted Treadmill Walking?	Lokomat	Stratified randomization 4 gait conditions of 5 min each: 1st condition at the beginning without Lokomat. Condition 2–4 with Lokomat with 3 levels of managers. Speed: 2 km/h; Conditions: Unassisted treadmill walking as well as during robot-assisted treadmill walking GF: 30, 60, and 100% BWS: 0	EEG BrainAmp DC & BrainVision Recorder, Brain Products GmbH, Germany; 32 electrodes Sampling rate: 1000 Hz Band pass: 0.5–100 Hz; Electrode placement: 10–20 system	Examination of ERSPs and PSDs during RATW at 30, 60, and 100% GF	No health impairments; Number of test persons: 11; Exclusion: 7; Male: 3; Female: 9; Age: 28.2 ± 4.0 years; Weight: 64,7 ± 7,7 Kg	Gait-related spectral modulations in the mu-, beta- and lower gamma bands above the SMC, related to certain phases of the gait cycle. Mu and beta rhythms were suppressed in the right primary sensory cortex during treadmill walking compared to 100% robot-assisted treadmill training, indicating significantly greater involvement of the sensorimotor area during treadmill walking compared to robot-assisted treadmill walking. Minor differences in the spectral performance of mu, beta and lower gamma bands between robotic treadmill walking with different guidance strengths.	High leadership strength and thus less active participation in the movement should be avoided during robot-supported treadmill training. This will optimize the participation of the sensorimotor cortex, which is known to be essential for motor learning.
Lapitskaya et al.,2011	Robotic gait training in patients with impaired consciousness due to severe traumatic brain injury	Lokomat	Prospective, controlled, non-randomized study. Single training session: Speed: 1.5 ± 0.1 km/h (patients), 1.61 ± 0.08 km/h (healthy subjects); Training time: 17.1 ± 1.3 min (patients), 17.15 ± 0.11 min (healthy volunteers); Walking distance: 427.3 ± 38.6m (patients), 451.36 ± 15.02m (healthy volunteers); conditions: Recovery phases and RAGT; GF: 100%; EEG recording in sitting position	EEG Nervous system (Taugagreining hf, Reykjavik, Island) 19 electrodes placement: Fz, Cz and Pz; ECI Electro-Cap SystemTM, International, Inc., Eaton, OH, United States; SEP measurement: ’VikingQuest’ (Viasys Healthcare, San Diego, CA, United States)	Sensory nerve tracts were evaluated with the help of sensory ERPs. Global DAR and the latency of the P300 component of the event-related potentials before and after a training session.	Patients with TBI disturbances of consciousness; Sample size: 12; Male: 9; Female: 3; Age: 40.8 ± 18.2 years; Control group: 14 healthy male subjects; Age: 47.3 ± 14.5 years	Basic measurement: Impaired SEPs in most patients and a significantly larger DAR in patients compared to healthy ones; After RAGT: Reduction of DAR in healthy subjects, but not in patients. No changes in P300 latency after training in patients or healthy subjects.	The study showed that robotic gait training induces measurable changes in the EEG performance spectrum in healthy individuals, while no changes were observed in patients with severe TBI. The absence of changes in the EEG power spectrum after RAGT in the patient may be an indicator of the severity of the injury.
Nakanishi et al.,2014	Rapid changes in arousal states of healthy volunteers during robot-assisted gait training: a quantitative time-series electroencephalography study	GAR	Conditions: Standing versus passive RAGT Standing: 30 s with eyes closed and 30 s with eyes open; RAGT: 6 min. at 3 conditions: (1) sinus wave noise stimulation, (2) verbal noise stimulation, (3) no noise stimulation; Speed: 0.11 m/s	EEG Polymate II AP216, TEAC, Tokyo, Japan Sampling rate: 1000 Hz; Electrode placement: 10–20 system	The PSD of the theta, alpha-1 and alpha-2 bands were calculated as indicators of objective drowsiness.	No health impairments; Sample size: 12; All male Age: 39.3 ± 1.8 years; Weight: 64.9 ± 2.3 kg; Body height: 168.4 ± 0.8 cm	Increase power density in theta (4.0–7.9 Hz) and alpha bands (ERS).	EEG-measured excitation values during RAGT decreased within a short time but can be restored and maintained by intermittent warning tone stimulation.
Seeber et al.,2013	Spatial-Spectral Identification of μ and β EEG Rhythm Sources During Robot-Assisted Walking	Lokomat	Conditions: 3 runs standing upright, 3 min each; 4 runs active walking, 6 min each	EEG 120 electrodes Sampling rate: 2.5 kHz; High pass: 0.1 Hz; Low pass: 1000 Hz; Electrode placement: 10–20 system	Power spectra (ERD); Functional brain topography	No health impairments; Sample size: 8; Male: 5; Female: 3; Age: 26.3 ± 3.5 years	Individual mu and beta ERD activities in SMC. The beta-ERD is more focal and consistent among the test persons in the foot area than the mu-ERD.	A method capable of considering individual slight differences in the rhythms mu and beta and locating the ERD activity of these rhythms at the cortical level. Maximum frequencies of ERD were successfully identified for each subject in the frequency range mu and beta. The resulting spectral peaks lead to mu and beta topographies for these frequencies.
Seeber et al.,2014	EEG beta suppression and low gamma modulation are different elements of human upright walking	Lokomat	Conditions: 4 runs active walking 6 min each, 3 runs upright standing 3 min each. Speed: 1.8 km/h - 2.2 km/h (constant, adapted to the test persons) BWS: <30% GF: 100%	EEG BrainAmp, Brain-products, Germany; 4× 32-channel amplifiers combined to 120 channels; Sampling rate: 2.5 kHz Band pass: 0.1–1000 Hz; Electrode placement: 10–20 system (EasyCap, Germany)	Amplitude Modulation & Power Spectra (ERD)	No health impairments; Sample size: 10; Male: 5; Female: 5; Age: 25.6 ± 3.5 years	During active walking, mu (10–12 Hz) and beta (18–30 Hz) oscillations were suppressed (ERD) compared to standing upright. Significant beta ERD was visible in 9/10 subjects in central sensomotoric areas. Low gamma (24–40 Hz) amplitude were modulated in relation to the gait cycle phase.	Persistent mu and beta ERD reflect a motion-related state change in cortical excitability, while gait-phase modulations in the lower gamma represent the motion sequence time during walking.
Seeber et al.,2015	High and low gamma EEG oscillations in central sensorimotor areas are conversely modulated during the human gait cycle	Lokomat	Randomized 8 runs RAGT 6 min each (4 with active walking and 4 with passive walking), 3 runs standing upright. 3 min rest between the runs. Speed: 1.8 km/h - 2.2 km/h; GF: 100%; BWS: <30%	BrainAmp amplifier, Brain-products, Germany; 4× 32 channel amplifiers combined to 120 channels. Sampling rate: 2.5 kHz; Band pass: 0.1–1000 Hz; Electrode placement: 10–20 system (EasyCap, Germany); Additional: EMG	Temporal dynamics of EEG oscillations in the source space by using time-frequency decomposition; Amplitude differences between walking and standing in mu (10–12 Hz), beta (18–30 Hz, gamma (60–80 Hz) and low gamma 24–40 Hz), high gamma (70–90 Hz)	No health impairments; Sample size: 10; Male: 5; Female: 5; Age: 25.6 ± 3.5 years right-handed	Increased gamma (60–80 Hz) amplitudes in central SMC and modulation of high gamma during walking compared to standing; High gamma and low gamma amplitudes are both modulated in relation to the gait cycle, but conversely to each other	Altered synchrony state during walking compared to standing due to static increase of gamma amplitudes; Dynamic, amplitude modulations at 70–90 Hz during gait cycle may reflect gait phase dependent interactions in locomotor network; Distinction of high and low gamma amplitudes in walking experiments due to its negative correlation
Villa-Parra et al.,2015	Toward a robotic knee exoskeleton control based on human motion intention through EEG and sEMGsignals	H2 Exoskelett Additional: UFES’s Smart Walker	Conditions: Sit down/stand up; Knee flexion/extension; 60 trials per 10 s, 3 min rest between conditions	EEG BrainNet BNT 36 electrodes; Additional sEMG	EEG: HMI analysis using: ERD/ERS & ERP (slow cortical potentials); sEMG: myoelectric pattern classification with regard to the lower limb	No health impairments; Sample size: 4; All male	Highest beta ERD in the range from 20 to 24 Hz; Highest beta ERS in the range from 16 to 22 Hz	Combination of EEG/sEMG signals can be used to define a control strategy for the robot system
Wagner et al.,2012	Level of participation in robotic-assisted treadmill walking modulates midline sensorimotor EEG rhythms in able-bodied subjects	Lokomat	Randomized 8 runs RAGT 6 min each (4 with active walking and 4 with passive walking), 3 runs standing upright. 3 min rest between the runs. Speed: 1.8 km/h - 2.2 km/h; GF: 100%; BWS: <30%	EEG BrainAmp DC and MR plus amplifier, Brain-products, Germany; 4×32 channel amplifiers combined to 120 channels. Sampling rate: 2.5 kHz; Band pass: 0.1–1000 Hz; Electrode placement: 10–20 system (EasyCap, Germany); Additional: EMG	Power spectra relating to the active and passive robot-supported gait	No health impairments; Sample size: 14; Exclusion: 1; Male: 8; Female: 7; Age: 22 to 28 (average: 24.3 ± 2.7) right-handed	Mu (8–12 Hz) and beta (18–21 Hz) rhythms are suppressed during active walking compared to passive walking.	Significant differences in cortical activation between active and passive robotic walking support the evaluation of brain monitoring techniques and brain-computer interface technologies to improve gait restoration therapies in a top-down approach.
Youssofzadeh et al.,2014	Directed neural connectivity changes in robot-assisted gait training: A partial Granger causality analysis	ALEX II on non-dominant leg (left)	Speed: 0.87 ± 0.15 m/s. 10 training paradigms Rest: 2 - 4 min. between trials Haptic and visual guidance: 100%.	EEG; g.tec’s; g.USBamp; 16 electrodes; Electrode placement: 10–20 system	PGC to elucidate the functional connectivity of EEG signals in RAGT; PSD for validity check	No health impairments; Sample size: 6 male subjects Age: 26.5 ± 6.5 years; Weight: 77.8 ± 9.7 kg; Body height: 1.79 ± 0.04 m	The results showed a strong causal interaction between the lateral motor cortical areas. A front-parietal connection was found in all robotic training units. After training a causal “top-down” cognitive control was found.	Causal ”top-down” cognition control indicates plasticity in connectivity in the respective brain regions.
Youssofzadeh et al.,2016	Directed Functional Connectivity in Fronto-Centroparietal Circuit Correlates with Motor Adaptation in Gait Training	ALEX II on dominant leg (right)	Conditions: Standing upright, walking unsupported on a treadmill, robot-assisted walking with and without the task of adjusting the changed footpath on a screen. 10 training paradigms Speed: 0.87 km/h ± 0.15 m/s	EEG; g.tec’s; g.USBamp; 16 electrodes; Sampling rate: 512 Hz; Electrode placement: 10–20 system; Additional: EMG	PGC to elucidate the functional connectivity of EEG signals in RAGT; PSD for validity check	No health impairments; Sample Size: 6 male subjects; Age: 26.5 ± 6.5 years; Weight: 77.8 ± 9.7 kg; Body height: 1.79 ± 0.04 m	PGC analysis showed improved connectivity near sensorimotor areas (C3, CP3) during standing while additional connectivity near central (CPz) and frontal (Fz) areas during walking compared to standing. Significant fronto-centroparietal causal effects both in training and after training. Strong correlations between kinematic errors and fronto-centroparietal connectivity during and after training. PSD analysis showed increase in α rhythms during standing, and theta and γ during walking.	Fronto-centroparietal connectivity is a potential neuromarker for motor learning and adaptation in RAGT.
Kim et al.,2016	Best facilitated cortical activation during different stepping, treadmill, and robot-assisted walking training paradigms and speeds: A functional near-infrared spectroscopy neuroimaging study	Lokomat	Randomized, based on a block design; 3 Conditions: Conventional walking, TW, RAGT; Speed: (1) self-selected, (2+3) 1,5, 2.0, 2.5, 3,0 km/h; GF: 100%. BWS: 50%	fNIRS 31-channels	Cerebral hemodynamic changes associated with cortical movement network regions in the primary SMC, PMC, SMA, PFC and SAC	No health impairments; Sample size: 14; Men: 8; Women: 6; Age: 30.06 ± 4.53 years right-handers	More global activation of the motion network (SMC, PMC, SMA) during RAGT compared to conventional and treadmill walking. Positive correlation of speed and activity of the movement network.	RAGT provides the best cortical activation associated with motor control.
Simis et al.,2018	Using Functional near Infrared Spectroscopy (fNIRS) to assess brain activity of spinal cord injury patient, during robot-assisted gait	Lokomat	Conditions: Standing (resting position) and walking in the Lokomat	fNIRS 32 optodes: 16 emitters, 16 detectors; Placement: 10–20 system	Cerebral hemodynamic changes in the motor cortex of both hemispheres. Relative change in concentration of oxy- and deoxyhemoglobin	Patients with spinal cord injury; Number of test persons: 3 patients	Two of the patients had an increased activation in M1 during the RAGT, compared to the standing position. One of the patients showed no changes in M1 brain activity.	fNIRS is suitable for measuring the brain activity of SCI patients during robotic walking. Results indicate an increased involvement of the motor cortical areas during walking.

*ALEX, active leg exoskeleton; BWS, body weight support; CSE, cortical spinal excitability; DAR, delta-alpha EEG power ratio; EGT, ekso-gait training; ERD, event-related desynchronization; ERS, event-related synchronization; ERSPs, event-related spectral perturbation; FPEC, frontoparietal effective connectivity; GAR, gait assistance robot; GF, guidance force; HMI, human-motion-intention; M1, primary motor cortex; OGT, over ground gait training; PFC, prefrontal cortex; PGC, time-domain partial Granger causality; PMC, primary motor cortex; PSD, power spectral density; RAGT, robot-assisted gait training; SAC, sensory association area; sEMG, surface electromyography; SEP, sensory evoked potentials; SMA, supplementary motor area; SMC, sensorimotor cortex; SMI, sensorimotor integration; TBI, traumatic brain injury; TMS, transcranial magnetic stimulation.*

## Review Criteria

**FIGURE 1:**
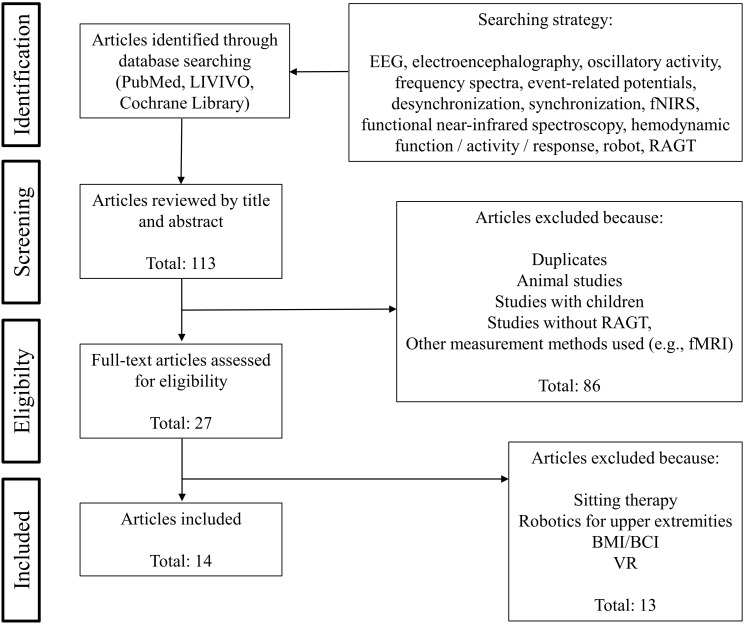
Flowchart of the article selection process.

The present mini review article focuses on studies addressing EEG or fNIRS during RAGT, thus investigating oscillatory and hemodynamic activity during robot-assisted gait were the decisive criterions. We followed the Preferred Reporting Items for Systematic Reviews statements (Moher et al., 2009) to identify and screen the articles. Figure 1 presents the search strategy as well as the selection criteria in detail. A total of 14 articles matched the inclusion criteria. All relevant data extracted and summarized from the screening process were shown in Table 1.

## Neural Correlates of RAGT

Based on 113 studies identified through database searching, 27 studies were deemed eligible for full-text assessment based on abstract review with 14 studies meeting our final inclusion criteria (see Figure 1). Twelve studies used EEG, two studies measured with fNIRS (see Table 1).

In EEG studies, the Lokomat (Hocoma, Switzerland) was used most frequently (Lapitskaya et al., 2011; Wagner et al., 2012; Seeber et al., 2013, 2014, 2015; Knaepen et al., 2015) whereas the active leg exoskeleton (ALEX; Youssofzadeh et al., 2014, 2016) was used in two studies and the gait assistance robot (GAR; Nakanishi et al., 2014), H2 exoskeleton (Villa-Parra et al., 2015; Contreras-Vidal et al., 2018) or the wearable Ekso^TM^ (Calabrò et al., 2018) in one study only. Changes in electrophysiological activity due to RAGT were analyzed by investigating either event-related potentials, power-spectral densities as well as coherence. Examinations were carried out either with healthy volunteers or in three studies with neurologically ill patients.

In healthy subjects, Wagner et al. (2012) investigated spectral patterns during active and passive robot-assisted walking and showed significantly suppressed mu (8–12 Hz) and beta (18–21 Hz) rhythms in central midline areas during active walking that also depend on gait cycle phases. These results have been underpinned by suppressed mu (10–12 Hz) and beta (18–30 Hz) oscillations (Seeber et al., 2013) as well as increased gamma amplitudes (24–40 Hz) during robot-assisted walking compared to standing (Seeber et al., 2015) or reduced power in alpha and beta bands during active participation due to less guidance force compared to complete guidance (Knaepen et al., 2015). Furthermore, low gamma (24–40 Hz; Wagner et al., 2012; Seeber et al., 2014) and high gamma (70–90 Hz) amplitudes were modulated depending on gait cycle phases, but conversely to each other (Seeber et al., 2015). Youssofzadeh et al. (2014, 2016) reported significant fronto-centroparietal connectivity during and after RAGT proposing potential neuromarkers for motor learning and adaptation in RAGT. Other investigations aimed to eliminate EEG artifacts by using REMOV, a method that combines various approaches and established ICA based routines to remove contaminations, thus providing a procedure to use EEG as an imaging technique during RAGT (Artoni et al., 2012). Clinical studies were conducted either with patients suffering from stroke (Calabrò et al., 2018; Contreras-Vidal et al., 2018) or traumatic brain injury (Lapitskaya et al., 2011). In a prospective, pre-post, randomized RAGT based clinical study with 40 stroke patients, improved frontoparietal effective connectivity (FPEC) after eight weeks of Ekso^TM^ gait training compared to conventional training was observed (Calabrò et al., 2018). Contreras-Vidal et al. (2018) showed changes in neuroelectric cortical activity patterns during post-stroke rehabilitation, thus, demonstrating the feasibility of decoding walking from brain activity.

In the two fNIRS studies, hemodynamic changes in response to RAGT were investigated in healthy subjects (Kim et al., 2016) as well as in patients with spinal cord injury (Simis et al., 2018). Kim et al. (2016) compared cortical activation during different stepping, treadmill, and RAGT paradigms and speeds. Cerebral hemodynamic changes were determined in cortical locomotor network areas. Elevated global locomotor network activation was observed during RAGT compared to stepping or treadmill walking, thus leading to the conclusion that RAGT facilitated greater cortical activation associated with locomotor control than without RAGT (Kim et al., 2016). The feasibility of fNIRS in patients suffering from spinal cord injury were shown by enhanced activation in motor cortex during RAGT compared to standing. This supports the assumption of enhanced involvement of motor cortical areas during RAGT (Simis et al., 2018).

In summary, the majority of EEG based studies demonstrated suppressed mu and beta oscillations while in fNIRS studies increased Hboxy changes in sensorimotor areas during RAGT were observed. Nevertheless, it must be noted that the studies differ significantly in terms of their methodology such as robotic device, EEG/fNIRS system, design, subjects and parameters that lead to heterogeneous results (see Table 1). To date, the complexity of our brain still leads to a limited understanding of the relation among brain activation and behavior as well as dysfunction and disorder. While measuring different perspectives of brain activity, the utilization of fused EEG-fNIRS helps to identify more features correlated with brain activation and brain connectivity in the complex process of gait. However, questions remain how the signals are related, and which advantages the fusion provides in terms of studying brain dysfunction due to gait disorders as well as neurophysiological changes during robotic gait rehabilitation?

## Fused EEG-fNIRS to Monitor Brain Activity

Both EEG and fNIRS are comparatively inexpensive and portable, they have unique, complementary advantages without interfering with each other, thus leading to an increased interest for fused EEG-fNIRS in rehabilitation research. The connection of the systems can be ensured either via wired trigger signals, which are split into various synchronized signals, or via connected networks. For the latter, the signals from different devices could be synchronized using systems (e.g., Lab Streaming Layer) where the networking and time-synchronization of the signals are handled to offer a uniform recording of measurement time series (Chadwick, 2018). Different approaches have been developed by taking advantage of simultaneous multimodal measurement.

First, fused EEG-fNIRS can be used for the investigation of how neuronal changes are related to neurovascular coupling. During a motor task, increased Hboxy concentration was accompanied by a decrease in Hbdeoxy and a decrease in alpha and beta power (Lachert et al., 2017). Koch et al. (2008) also reported a correlation between high individual alpha frequency (IAF) and low Hboxy response (Koch et al., 2008). Furthermore, Berger et al. (2018) extended the relation by demonstrating an increase of EEG alpha power following 10 and 20 Hz transcranial alternating current stimulation (tACS), which was accompanied by a decrease in Hboxy (Berger et al., 2018). Based on previous studies investigating neural correlates of walking or RAGT, it can be expected that suppressed alpha and beta oscillations in the motor cortical network are accompanied by increases in Hboxy. However, numerous questions regarding the correlation of neuronal activities and vascular changes (e.g., co-localization / time lag of the correlated changes or consistency of changes in connectivity; Functional Brain Imaging with EEG and fNIRS, o.d.) remain.

Second, fused EEG-fNIRS provides detailed spatio-temporal information on neurophysiological activity through different strengths and weaknesses of EEG and fNIRS, thus leading in improved distinctions of conditions such as rest and task (Leamy et al., 2011; Fazli et al., 2012; Khan et al., 2018). Leamy et al. (2011) investigated hemodynamic and electrical responses during motor imagery (MI) and rest periods. Classification of 2-dimensional EEG feature spaces (change in mu and beta power from rest to MI) and fNIRS feature spaces (change in average amplitude of Hboxy and Hbdeoxy from rest to MI) was performed using linear discriminant analysis (LDA), at first separately and in combination afterward. By employing a supplemental measurement modality through combining EEG-fNIRS, more information about the underlying neurovascular relationship can be achieved (Leamy et al., 2011). If the classification of fNIRS features can complement EEG classifications were also examined in a real-time sensor motor-rhythm based brain-computer interface (BCI) paradigm with executed movements and MI. By using LDA, fused EEG-fNIRS provide complementary spatio-temporal information with a significantly improved classification accuracy (Fazli et al., 2012). Furthermore, it would be possible to use data by one technique to reduce artifacts of the other due to the independence between electrical and optical measurements but up to date, these possibilities are not yet sufficiently proven. By the fact that fNIRS is limited due to the long-term delay of the hemodynamic response (Fazli et al., 2012) and requires a minimum distance between sources and detectors that detectors are not suffering from “source blinding”, improving fNIRS through supplementing EEG data might be more useful than adding fNIRS to EEG (Leamy et al., 2011).

In summary, the independence between electrical and optical measurements can be used advantageously to (1) obtain detailed spatio-temporal information, (2) improve classification accuracy and (3) reduce effects of movements artifacts with the aim to improve the reliability and robustness of signal interpretation (Shin et al., 2018). Nevertheless, the relation of EEG and fNIRS is not yet fully understood (Leamy et al., 2011) and challenges of the combined EEG-fNIRS approach arise. For instance, electrical and hemodynamic signals are not necessarily coupled. On one side, physiological process (e.g., neurotransmitter synthesis) can cause hemodynamic changes without electrophysiological activity. On the other side, changes in metabolic activity may not be detectable if EEG activity is transient (Yuan et al., 2014). Furthermore, EEG and fNIRS measurements make great demands on study designs due to the susceptibility to motion and light interference (Vitorio et al., 2017) which is of high relevance especially for clinical studies. In addition, there is no uniform, standardized procedure due to many different methods regarding signal processing and analysis which makes the comparability and interpretation difficult. According to this, the combination of EEG and fNIRS is very complex and still in its infancy despite the technical progress.

However, based on the knowledge that multi-modal measurement has the capacity to analyze neurovascular coupling more accurately, fused EEG-fNIRS might be beneficial to basic neuroscientific research as well as clinical applications (Muthalib et al., 2013).

## Future Prospects for Fused EEG-fNIRS in RAGT

Regarding RAGT, fused EEG-fNIRS provides a detailed insight into how locomotor control and gait recovery is characterized by brain signals. On one side, the understanding of the underlying mechanisms of RAGT might be a further step toward scientific-based evidence for improved gait recovery. Based on brain signals, optimal training parameters and settings (e.g., BWS, GF; Knaepen et al., 2015) as well as augmented feedback (Wagner et al., 2014; Calabrò et al., 2017) can be determined for individualized gait therapy protocols. Wagner et al. (2014) examined the impact of visual feedback on EEG pattern during RAGT and presented that movement related interactive feedback in virtual environment (VE) significantly increase brain activity in premotor and parietal areas due to motor planning and visuomotor processes compared to movement unrelated feedback (Wagner et al., 2014). Thus, robot-assisted gait rehabilitation can be further improved, for example by selecting specific feedback based on the underlying network activity in order to promote individuals voluntary drive which is crucial for motor learning.

**FIGURE 2:**
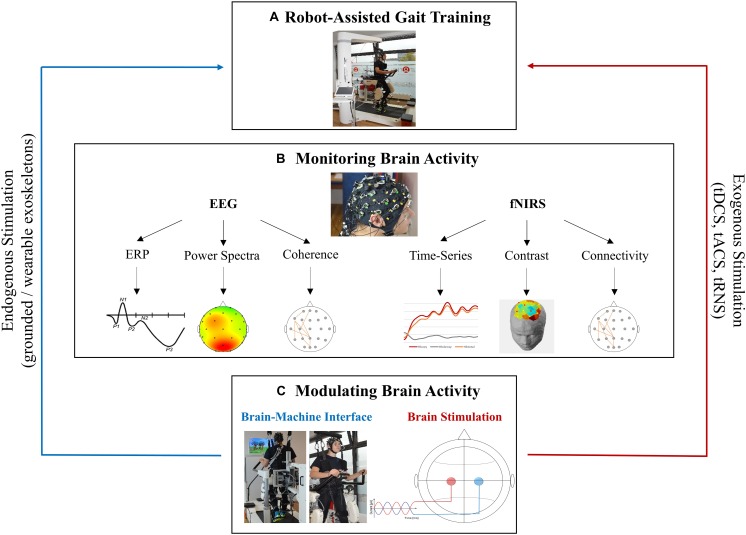
Future perspectives for fused EEG-fNIRS in robotic gait rehabilitation. **(A)** Robotic device Lokomat (Hocoma, Switzerland) for RAGT. **(B)** EEG and fNIRS provide the monitoring of neurophysiological processes occurring during RAGT by measuring different perspectives of brain activity. Electrophysiological (EEG) and haemodynamic (fNIRS) signals can be processed in various ways. Data from each electrode/channel can be extracted over time (e.g., ERPs/time-series), as an average over time (e.g., power spectra/contrasts) or they can be correlated to represent functional connectivity (e.g., coherence/connectivity). **(C)** Brain activity measured by EEG or fNIRS serves as a basis for brain modulation such as BCI/BMI which may be a form of endogenous brain stimulation as well as for brain stimulation techniques were a weak current is applied through the skull to modulate brain activity. Subjects appearing in the figures provided informed written consent to the publication of identifying images in an online open access publication.

On other side, EEG-fNIRS signals can be used to expand and integrate further approaches such as EEG/fNIRS controlled gait therapy in brain machine interface (BMI) paradigms or non-invasive brain stimulation (NIBS) during RAGT that are guided by oscillatory or hemodynamic activity (see Figure 2).

In BMI, cortical activity associated with movements were identified and used to directly control external devices (García-Cossio et al., 2015; Donati et al., 2016; Teo et al., 2016). Donati et al. (2016) investigated the effects of long-term training with a BMI-based gait protocol on motor recovery and restoring mobility in paraplegic patients. Eight chronic paralyzed patients performed a BMI neurorehabilitation paradigm including virtual reality training, enriched visual-tactile feedback, and walking with EEG-controlled robotic devices. After 12 months of training, paraplegic patients improved their somatic sensation and walking abilities. It was hypothesized that this unprecedented neurological recovery resulted from both cortical and spinal cord plasticity triggered by long-term training with BMI (Donati et al., 2016). For the future of non-invasive, portable, and wearable BMIs, researchers suggest the use of hybrid EEG-fNIRS systems, as they have been shown to be superior to the use of EEG-BCIs and fNIRS-BCIs alone (Fazli et al., 2012; Khan et al., 2014; Koo et al., 2015; Naseer and Hong, 2015).

Another future prospect could include the feedback of cortical activation pattern measured by fused EEG-fNIRS that is used to identify regions of hypo- or hyperactivity to guide NIBS protocols (Teo et al., 2016; Berger et al., 2018). Transcranial electrical stimulation (tES) is regarded as one of the most well-known forms of NIBS who’s primarily researched modalities are transcranial direct current stimulation (tDCS) and transcranial alternating current stimulation (tACS; Paulus, 2011; Yavari et al., 2018). By applying weak current through the scalp, tES represent promising tools for the induction of acute or long-lasting effects on excitability or brain network dynamics, thus investigating the causal relationship between brain activity, motor functions (Yavari et al., 2018) and potentially enhancing motor learning processes (Reis and Fritsch, 2011; Sugata et al., 2018). tES can be set up as portable and wireless systems, thus having complimentary capabilities as well as EEG and fNIRS (McKendrick et al., 2015). In this way, combining tES with fused EEG-fNIRS is suitable for modulating pathological brain pattern to enhance neuroplasticity during RAGT, thereby representing an avenue for clinical applications (McKendrick et al., 2015). For example, fused EEG/fNIRS allow the identification of hypo- or hyperactivity accompanied by gait disorders that might help to determine and guide brain stimulation protocols. Thus, tES may be applied during RAGT to modulate neuronal networks supporting gait rehabilitation (Teo et al., 2016). Closed-loop tES during RAGT as well as BMI guided by fused EEG-fNIRS represent two approaches for the implementation of individualized neurorehabilitation which is essential for success in motor recovery (McKendrick et al., 2015).

## Conclusion

EEG and fNIRS measure different perspectives of brain activity. Therefore, fused EEG-fNIRS provide detailed spatio-temporal information correlated with brain activity and connectivity. Based on the reviewed studies investigating brain activity following RAGT as well as studies using multi-modal approaches, we conclude that fused EEG-fNIRS has the potential to characterize the complex neurovascular coupling mechanisms associated with gait disorders due to neurological diseases as well as robotic rehabilitation in more detail than by using one modality alone. Thus, neuroplastic changes due to RAGT that were measured by fused EEG-fNIRS might contribute to scientifically prove the effectiveness of RAGT. Furthermore, revealing brain activity underlying RAGT is essential for the adjustment of therapy protocols and the guidance of further interventions such as BMI or NIBS. Fused EEG-fNIRS approaches the goal of individualized, closed-loop RAGT that optimizes the outcome and efficacy of gait recovery, thereby fostering independent living and improved quality of life for neurological patients. Despite technical advances (e.g., signal processing, data synchronization), fused EEG-fNIRS remains complex and poses challenges that require further innovations to determine the potential during RAGT in clinical and in non-clinical environment.

## Author Contributions

AB primarily contributed to the conceptualization of the review, performed the literature research, interpreted the data, wrote the manuscript, approved the final version of the manuscript, and acted as corresponding author. SM contributed to the literature research and wrote a part of the manuscript. FH, FS, and MD substantially contributed to the data interpretation and critically revised the manuscript.

## Conflict of Interest Statement

The authors declare that the research was conducted in the absence of any commercial or financial relationships that could be construed as a potential conflict of interest.
